# Proinflammatory Pathways Are Activated in the Human Q344X Rhodopsin Knock-In Mouse Model of Retinitis Pigmentosa

**DOI:** 10.3390/biom11081163

**Published:** 2021-08-06

**Authors:** T.J. Hollingsworth, Meredith G. Hubbard, Hailey J. Levi, William White, Xiangdi Wang, Raven Simpson, Monica M. Jablonski, Alecia K. Gross

**Affiliations:** 1 Department of Ophthalmology, Hamilton Eye Institute, College of Medicine, University of Tennessee Health Science Center, Memphis, TN 38163, USA; thollin1@uthsc.edu (T.J.H.); Wilawhit@uthsc.edu (W.W.); xwang17@uthsc.edu (X.W.); rdavi122@uthsc.edu (R.S.); mjablon1@uthsc.edu (M.M.J.); 2 Department of Neurobiology, Evelyn F. McKnight Brain Institute, University of Alabama at Birmingham, Birmingham, AL 35294, USA; hubbardm@uab.edu (M.G.H.); hlevi001@uab.edu (H.J.L.)

**Keywords:** retinal degeneration, retinitis pigmentosa, rhodopsin, inflammation, microglia, TNFα, NF-κB, JAK/STAT, NLRP3

## Abstract

Retinitis pigmentosa (RP) is a hereditary disease of the retina that results in complete blindness. Currently, there are very few treatments for the disease and those that exist work only for the recessively inherited forms. To better understand the pathogenesis of RP, multiple mouse models have been generated bearing mutations found in human patients including the human Q344X rhodopsin knock-in mouse. In recent years, the immune system was shown to play an increasingly important role in RP degeneration. By way of electroretinography, optical coherence tomography, funduscopy, fluorescein angiography, and fluorescent immunohistochemistry, we show degenerative and vascular phenotypes, microglial activation, photoreceptor phagocytosis, and upregulation of proinflammatory pathway proteins in the retinas of the human Q344X rhodopsin knock-in mouse. We also show that an FDA-approved pharmacological agent indicated for the treatment of rheumatoid arthritis is able to halt activation of pro-inflammatory signaling in cultured retinal cells, setting the stage for pre-clinical trials using these mice to inhibit proinflammatory signaling in an attempt to preserve vision. We conclude from this work that pro- and autoinflammatory upregulation likely act to enhance the progression of the degenerative phenotype of rhodopsin Q344X-mediated RP and that inhibition of these pathways may lead to longer-lasting vision in not only the Q344X rhodopsin knock-in mice, but humans as well.

## 1. Introduction

Retinitis pigmentosa (RP) is a hereditary retinal disease which causes degeneration due to the progressive loss of rod photoreceptors primarily by way of apoptotic mechanisms [1]. Ultimately, the cone photoreceptors die as well, leaving the patient blind. RP afflicts approximately 1 in every 4000 individuals and presents with high heterogeneity, making the disease difficult to treat or cure [1]. RP can be passed down to progeny through all modes of inheritance including autosomal dominant and recessive (adRP, arRP), X-linked and mitochondrial [1]. Mutations in rhodopsin are the leading cause of adRP cases, with approximately 10% and 30% of all RP and adRP cases, respectively, caused by rhodopsin mutations. Of all rhodopsin-mediated adRP cases, the most severe forms tend to be caused by mutations affecting the carboxyl-terminus of the protein. Among these, two of the most severe mutations are the Q344X and X349E mutant rhodopsins. These mutations cause mislocalization of both the mutant and wild-type (WT) rhodopsin to the plasma membrane of the rod inner segment (RIS), nuclear region and synapse [1,2,3,4,5]. Previously, we have reported on the X349E rhodopsin mutant by generating a knock-in mouse bearing the read-through mutation. This mouse model develops early onset and rapidly progressing retinal degeneration [2,3]. It was previously thought that the mutant protein resulting from the genetic mutation was sufficient to cause the degenerative phenotype observed in patients by causing protein mislocalization, endoplasmic reticulum stress, loss or gain-of-function, etc. [1,2]; however, it is now known that other endogenous factors play major roles in disease pathogenesis, specifically the immune system. Our groups and others have shown that while the mutated protein itself is sufficient to initiate retinal degeneration through one or more of many possible mechanisms, the establishment of a proinflammatory microenvironment by way of microglial activation and cytokine release-induced positive feedback assists in progressing the degeneration beyond that of the initial insult [3,6,7,8,9,10]. Thus, treatments for RP using immunomodulatory therapeutics may have the ability to prolong photoreceptor life and function, preserving vision for the patient beyond what is possible if left untreated; however, these treatments, in conjunction with other treatments such as gene therapy, could have the potential for life-lasting vision preservation and restoration.

In addition to the X349E rhodopsin mouse model, a Q344X knock-in mouse has also been generated [11,12]. This Q344X rhodopsin knock-in mouse expresses the human Q344X rhodopsin gene; however, due to the insertion of a LoxP site in the upstream promoter region used to excise the hypoxanthine phosphoribosyltransferase (HPRT) minigene used for positive selection, the Q344X gene is expressed at lower levels than the WT gene (~10% of normal expression per allele) [12]. Due to this lack of normal levels of Q344X expression, heterozygote mice do not degenerate during the time course of this study and were excluded from data collection. To date, no study of inflammatory pathways in this mouse model has been performed and thus it was our purpose to examine the human Q344X rhodopsin knock-in mouse retina for degenerative and/or proinflammatory phenotypes compared with phenotypes previously observed in similar models. These include Müller cell hypertrophy, photoreceptor apoptosis and microglial migration and phagocytosis, as well as proinflammatory pathway activation, specifically the Janus kinase/Signal transducer and activator of transcription (JAK/STAT) pathway, tumor necrosis factor alpha (TNFα) signaling, nuclear factor kappa B (NF-κB) and OD-, LRR- and pyrin domain-containing protein 3 (NLRP3) inflammasome activation. We also demonstrate, through the use of fluorescent immunocytochemistry (fICC), the ability to inhibit the JAK/STAT pathway (a highly enriched pathway observed in the retinas of these mice) by pharmaceutical means. This sets the stage for future pre-clinical studies using pharmacological intervention to ameliorate the degenerative phenotypes observed in these animals. 

## 2. Materials and Methods

### 2.1. Q344X Rhodopsin Knock-In Mouse Model

Q344X rhodopsin knock-in mice (C57B/6) were obtained from Dr. T.G. Wensel (Baylor College of Medicine, Houston, Texas, TX, USA) and were described previously [12]. In brief, the mouse rhodopsin gene was substituted with a mutant human rhodopsin–EGFP fusion protein that additionally went under site-directed mutagenesis to replace a single nucleotide to change the codon at amino acid position 344 from glutamine (CAG) to a stop codon (TAG). To make these mice, Sandoval and colleagues electroporated mouse embryonic stem cells with the construct and these cells were negatively selected for using TK and positively selected for using HPRT. After selection, the ES cells were inserted into a blastocyst and a pseudopregnant female used to house the embryos. The HPRT mini-gene was excised by Cre recombinase using LoxP sites flanking the mini-gene by breeding the offspring to mice expressing Cre under the EIIA promoter. Due to the presence of the remaining LoxP site in the upstream promoter region, these animals express the Q344X rhodopsin at ~10% of normal levels per allele.

### 2.2. Electroretinographic (ERG) Analysis of the Q344X Rhodopsin Knock-In Mouse Retina

WT and Q344X mice at 3, 6, 9, 12, 15, 18 and 24 weeks of age of both sexes were dark adapted overnight then anesthetized using ketamine/xylazine (71.42 mg/kg ketamine/14.3 mg/kg xylazine in PBS, pH7.4) and pupils dilated using a mixture of 0.5% phenylephrine HCl, 0.5% proparacaine HCl and 1% tropicamide. ERG recordings were collected using the LKC UTAS Visual Electrodiagnostic Testing system (Gaithersburg, MD, USA) and silver-embedded thread electrodes in contact with the corneal surface with 2.5% hypromellose ophthalmic solution (GONIOVISC). Scotopic ERG was measured over a range of flash intensities (0.025–79.1 cd-s/m^2^). Photopic ERG was measured after a 10 min exposure to 25 cd.s/m^2^ background light followed by a range of flash intensities. The *a*-wave amplitude was measured from baseline to trough and the *b*-wave amplitude was measured from base to peak at each flash intensity. Latencies represent time (ms) to peak or trough. Data were compiled in Microsoft Excel and graphed in GraphPad Prism. Due to space concerns, though ERGs were performed at all ages from 3, 6, 9, 12, 15, 18 and 24 weeks, only the ages corresponding to the histological imaging are shown graphically in this manuscript.

### 2.3. Examining the Q344X Rhodopsin Knock-In Mouse Retina for Histological Changes Relative to WT by Optical Coherence Tomography (OCT)

WT and Q344X rhodopsin knock-in mice at 3, 9 and 21 weeks of age of both sexes were anesthetized using ketamine/xylazine (71.42 mg/kg ketamine/14.3 mg/kg xylazine in PBS, pH 7.4) and pupils dilated using a mixture of 0.5% phenylephrine HCl, 0.5% proparacaine HCl and 1% tropicamide. To keep the eyes lubricated and maintain corneal clarity, artificial tears (Systane Original) were applied when needed. The mice were subsequently examined by OCT using a Bioptigen 840 nm Spectral Domain-OCT (Bioptigen, Durham, NC, USA) through the optic nerve head with the purpose of measuring total retinal and ONL thicknesses and to assess for any laminar abnormalities that could be attributed to RP. Mice were tested at alternating ages to those tested for ERGs for the general purpose of in vivo observation of the degenerative phenotype to confirm what is observed histologically.

### 2.4. Assessing the Q344X Rhodopsin Knock-In Mouse Retina for Pigmentary and Vascular Anomalies Associated with RP

WT and Q344X rhodopsin knock-in mice at 6 and 15 weeks of age were anesthetized using ketamine/xylazine (71.42 mg/kg ketamine/14.3 mg/kg xylazine in PBS, pH7.4) and eyes dilated using tropicamide. The animals were then intraperitoneally injected with 100 μL of 4% fluorescein and subsequently imaged with either white light (funduscopy for bone spicule pigmentation) or 488 nm light (fluorescein angiography for vessel attenuation, neovascularization and hemorrhage) emitted from an Eyemera Fundus Camera (IIScience, 3003 N 1st St., San Jose, CA, USA). The ages chosen for these mice were selected based on our histological data showing the photoreceptor degeneration was initiated between 3 and 6 weeks (retinas look similar in thickness at 3 weeks of age but ONL thickness was reduced by 6 weeks) and has progressed to an essentially rod-free retina (reached at approximately 24 weeks of age) by 15 to 18 weeks of age.

### 2.5. Western Blot Analysis of Retinal Expression of Glial Hypertrophy, Microglial, and Proinflammatory Signaling Pathway Proteins

Retinas from Q344X mice at 6 weeks of age were extracted, sonicated in RIPA buffer with protease inhibitors, centrifuged to pellet cell debris and lysate electrophoresed on a 12% polyacrylamide gel with 4% stacking gel. Gels were stacked at 80 V for 20 min and resolved at 150 V for 1.5 h. Gels were then transferred to PVDF membranes and probed for GFAP, GS, IBA1, NF-κB, NLRP3, TNFα, STAT3, pSTAT3, SOCS3, and PKCα antibodies at 1:1000 dilutions (Table 1) overnight at 4 °C to test for antibody specificity. After washing blots free of primary antibodies 3 times in TBST for 15 min each, blots were then probed using secondary antibodies against mouse, rabbit, or rat IgGs conjugated to AlexaFluor680 or AlexaFluor800 (ThermoFisher; 168 Third Avenue, Waltham, MA, USA) at 1:10,000 dilutions for 1 h at room temperature (RT). Blots were subsequently washed in TBST 4 times for 15 min each and imaged using a LI-COR Odyssey Fc NIR Imaging System (LI-COR, 4647 Superior Street, Lincoln, Nebraska, NE, USA).

### 2.6. Labeling for Glial Hypertrophy, Apoptotic Cells, Microglia, and Proinflammatory Signaling Pathway Proteins in the Q344X Rhodopsin Knock-In Mouse Retina by Fluorescent Immunohistochemistry (fIHC) and TUNEL

Whole eyes from WT and Q344X rhodopsin knock-in mice at 3, 6, 9, 12, 15, 18, 21 and 24 weeks of age were enucleated and fixed in 4% paraformaldehyde in PBS, pH 7.4 overnight at 4 °C. Fixation was quenched in 100 mM Tris in PBS, pH 7.4 for 10 min at room temperature and subsequently washed in PBS. Eyes were dehydrated with 30 min incubations in a graded ethanol series (50%, 70%, 85%, 95% and 100%) then cleared via a 30 min incubation in graded xylenes (2:1, 1:1, and 1:2 ethanol: xylenes), and two 30 min incubations in 100% xylenes. Eyes were then infiltrated with paraffin using a graded paraffin series with 30 min incubations in 2:1, 1:1, and 1:2 xylenes: paraffin and two subsequent 1 h incubations in 100% paraffin. Paraffin-embedded tissue was then sectioned at 8 µm or 15 μm and sections deparaffinized and rehydrated, treated using heat-mediated antigen retrieval by heating slides at 95 °C in sodium citrate buffer (10 mM sodium citrate, 0.05% Tween-20, pH 6.0) for 1 h, washed in PBS twice and subsequently blocked in 10% goat serum/5% BSA/0.5% TritonX-100 in PBS for 30 min at RT. Primary antibodies against markers for glial stress/hypertrophy, microglia, and proinflammatory signaling pathways were then applied at recommended dilutions (Table 1) and incubated overnight at 4 °C. Slides were then washed in PBS, pH 7.4 three times for 10 min each. Post-washing, slides were then incubated in secondary antibodies conjugated to either AlexaFluor488, AlexaFluor568, or AlexaFluor647 (ThermoFisher; 168 Third Avenue, Waltham, MA, USA) at 1:400 dilutions for 1 h and nuclei stained using DAPI. For phosphorylated STAT3 (pSTAT3), tyramide signal amplification (TSA) was performed using the TSA with SuperBoost kit (ThermoFisher; 168 Third Avenue, Waltham, MA, USA) following the manufacturer’s instructions. Slides were then washed in PBS, pH 7.4 four times and mounted using Prolong Diamond Antifade mountant (ThermoFisher; 168 Third Avenue, Waltham, MA, USA). For TUNEL labeling, the Click-It Plus TUNEL kit (ThermoFisher; 168 Third Avenue, Waltham, MA, USA) was used to label apoptotic nuclei with AlexFluor647 following the manufacturer’s instructions. After drying overnight, sections were imaged using a Zeiss 710 laser scanning confocal microscope using a 40X objective with 1.3 numerical aperture and a 63X objective with 1.4 numerical aperture and 1.5X zoom. Although the expression of all proinflammatory markers increased with age, it should be noted that due to space restraints, images taken from ages with the best representative images of changes in expression are presented.

### 2.7. Analysis of JAK/STAT Activation and Pharmacological Inhibition in Rat Müller Cells (rMC-1) Using FICC

rMC-1 cells were grown on poly-L-ornithine/laminin coated glass bottom plates to ~95% confluency and subsequently treated with Leukemia inhibitory factor (LIF, activator of JAK/STAT pathway) lacking or in the presence of upadacitinib (UPA, JAK inhibitor; MilliporeSigma; 3050 Spruce St., St. Louis, MO, USA) for 1 h. Multiple concentrations of UPA were tested and a dose of 500 nM was chosen for inhibition of both JAK1 and JAK2, both of which activate STAT3 in pro-inflammatory conditions [13]. 500 nM is approximately 4 times the concentration of the IC_50_ for JAK2, which we expect to allow for sufficient delivery of a relevant dose of UPA if given in the form of a topical treatment. Cells were washed in PBS, pH 7.4, fixed in 4% paraformaldehyde in PBS, pH 7.4 for 10 min at room temperature, and fICC labeled for STAT3 and pSTAT3 using the same antibodies as for fIHC. Cells were imaged using a Zeiss 710 laser scanning confocal microscope using a 40X objective with 1.3 numerical aperture.

## 3. Results

### 3.1. Q344X Rhodopsin Knock-In Mice Experience Functional Deficits by ERG

**Figure 1:**
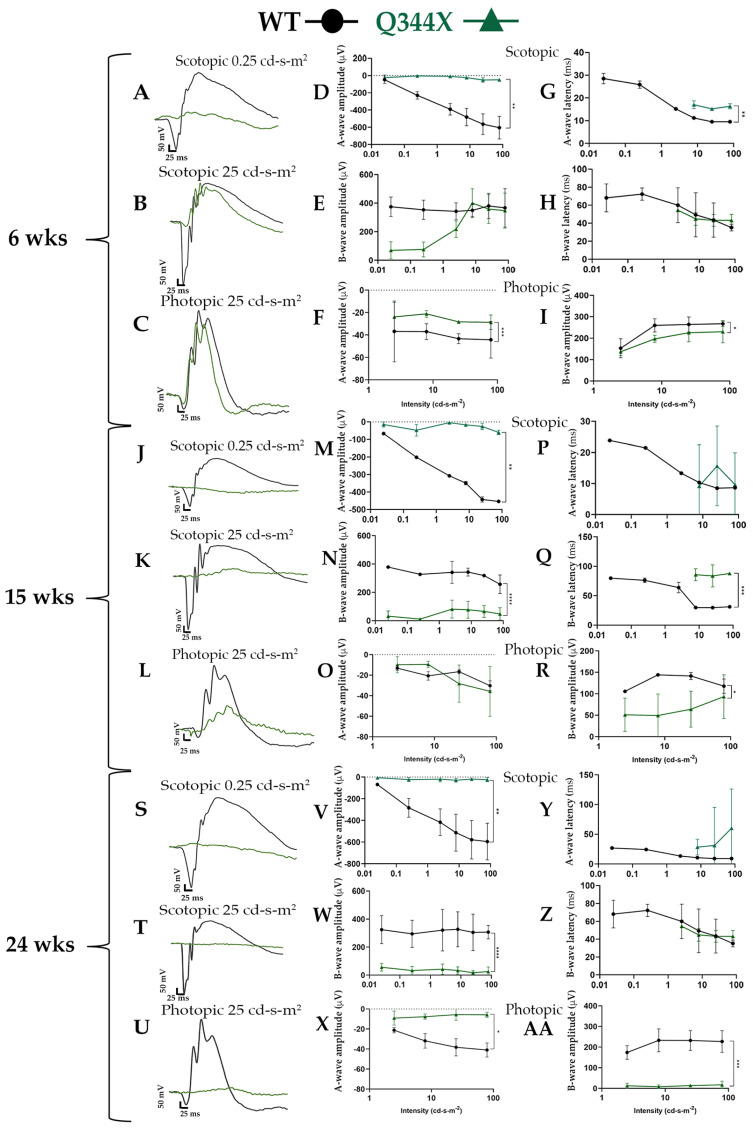
Q344X rhodopsin knock-in mice elicit minimal rod cell responses by ERG. (**A**–**C**, **J**–**L**, **S**–**U**) Overlaid representative scotopic and photopic ERG traces at low and high flash intensities (0.25 and 25 cd-s-m2) from dark and light-adapted animals at 6 weeks (**A**–**C**), 15 weeks (**J**–**L**), and 24 weeks **(S**–**U)**: WT (black) and Q344X (green). (**D**–**AA**) Summary data averaged across WT (black circles) and Q344X (green triangles). A-wave and b-wave amplitudes were measured from base line to trough or peak respectively for each flash intensity for 6 weeks (**D**–**F**), 15 weeks (**M**–**O**), and 24 weeks (**V**–**X**). Latencies represent time to peak or trough for each flash intensity for 6 weeks (**G**–**I**), 15 weeks (**P**–**R**), and 24 weeks (**Y**–**AA**). Q344X mice demonstrated significantly decreased a-wave amplitude compared to WT between 3 and 6 weeks. A measurable b-wave was present in scotopic tracings of young Q344X mice; however, the tracing correlated with b-waves in photopic tracing. At 24 weeks, Q344X mice responses were flatline (*n* = 3-4 per group; mean ± SD; * *p* = 0.02, ** *p* < 0.05, *** *p* < 0.0005, **** *p* < 0.0001).

As the retina degenerates, the electrical signals being generated by the retinal neurons decrease over time. Because of this, we tested the neuronal function of the retinas of Q344X rhodopsin knock-in mice at various ages. Rod and cone photoreceptor function was monitored using ERG in 6, 15 and 24 week old WT and Q344X rhodopsin knock-in mice (*n* = 3–4, Figure 1). At 6 weeks, Q344X mice had severely decreased a-wave amplitudes in the scotopic intensity range compared to WT (*p* = 0.005). These mice displayed a measurable b-wave; however, when compared to photopic traces, these waveforms were similar to the cone response. At 15 weeks, Q344X mice have severely reduced a-waves (*p* < 0.05) and slight, measurable b-waves which are statistically decreased compared WT (*p* < 0.0001). WT mice continue to have similar a- and b-waves through 24 weeks. At 24 weeks, both a- and b-wave amplitudes are severely reduced in all tracings of Q344X mice (*p* = 0.0005). A- and b-wave latency was measured by calculating the time (ms) to trough or peak, respectively. Q344X mice displayed consistently longer a-wave latencies compared to WT while b-wave latencies remained mostly consistent throughout groups and ages with 15 weeks showing an increased latency compared to WT.

### 3.2. Retinas from Q344X Rhodopsin Knock-In Mice Display Degenerative RP Phenotypes by OCT and Funduscopy

**Figure 2:**
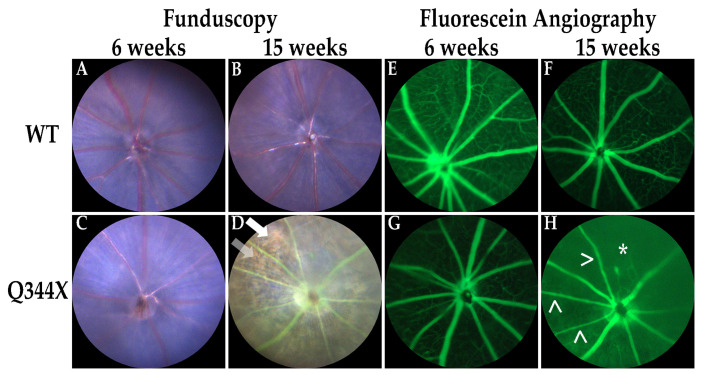
Q344X rhodopsin knock-in mouse retinas exhibit hallmark phenotypes of RP by funduscopy and fluorescein angiography. WT and Q344X mouse retinas at 6 and 15 weeks of age were imaged by funduscopy using white light (**A**–**D**) or 488 nm light post-fluorescein injection (**E**–**H**). Arrowheads (>) indicate attenuated vessels. Arrows indicates bone-spicule pigmentation. Asterisk (*) indicates loss of vessels.

Retinal degenerations such as RP result in thinning of the outer retina and a resultant bone spicule pigmentation pattern caused by the retinal pigment epithelium (RPE) being observable through the thinning retina [1,2]. In addition, the vasculature of the retina becomes augmented causing attenuation of the retinal vessels and/or an increase in the tortuosity of vessels [3,14]. To discern these phenotypes in vivo, OCT and fundoscopy with accompanying fluorescein angiography were performed. As observed in previous studies of this model [12], the retinas of human Q344X rhodopsin knock-in mice rapidly degenerate with a decline in ONL thickness by 6 weeks and to almost no rod photoreceptors by 15 weeks (Supplementary Materials Figure S1). These animals also demonstrate sharply attenuated and tortuous retinal vessels and a highly pronounced bone spicule pigmentation pattern consistent with RP (Figure 2).

### 3.3. Western Blot Analysis of FIHC-Probed Proteins

In order to test for antibody specificity in fIHC images, Western blots were performed using antibodies for fIHC (Supplementary Materials Figure S2). All antibodies displayed banding patterns expected based on calculated molecular weights. SOCS3 displayed two bands when one was expected; however, this double banding pattern is explained by an N-terminally truncated isoform resulting from translation starting at a second methionine at codon 12 observed under stressed conditions [15].

### 3.4. Upregulation of Glial Fibrillary Acidic Protein (GFAP) Expression in the Q344X Rhodopsin Knock-In Mouse Retina

**Figure 3:**
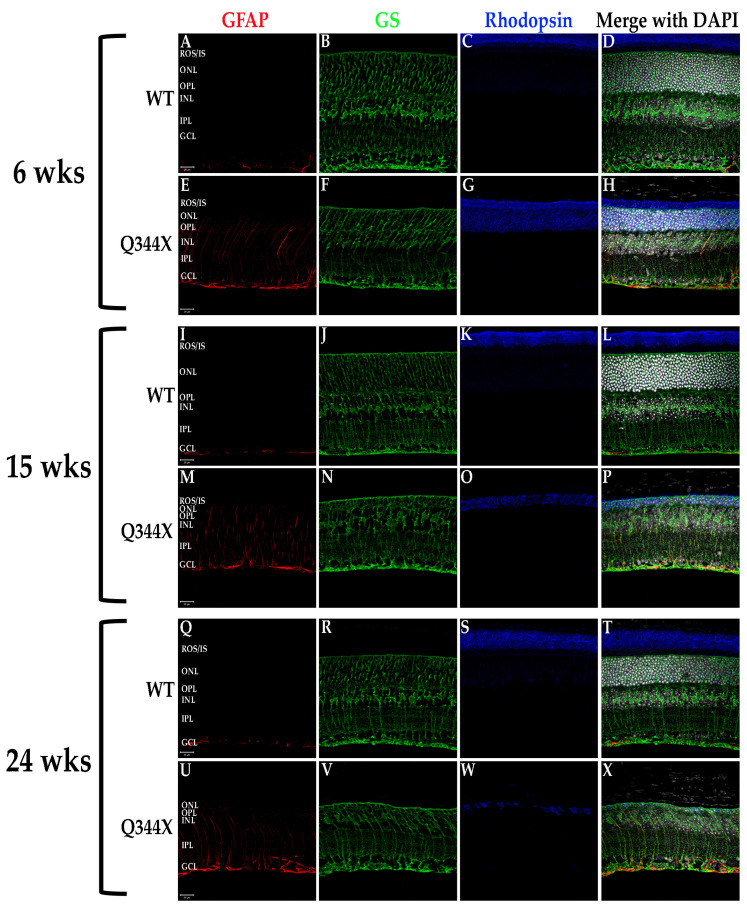
Müller cells exhibit GFAP upregulation/hypertrophy in the Q344X rhodopsin knock-in mouse retina. WT (**A**–**D**, **I**–**L**, **Q**–**T**) and Q344X (**E**–**H**, **M**–**P**, **U**–**X**) mouse retinal sections at 6, 15, and 24 weeks of age were immunolabeled for GFAP (red), GS (green) and rhodopsin (blue). Nuclei were labeled with DAPI (white). ROS/IS, rod outer segments/ inner segments; ONL, outer nuclear layer; OPL, outer plexiform layer; INL, inner nuclear layer; IPL, inner plexiform layer; GCL, ganglion cell layer. Scale bars = 20 µm.

All retinal degenerations tend toward a strong enrichment of the glial protein GFAP in Müller cells spanning the retina [16]. This GFAP increase is associated with hypertrophy of the Müller cells. As such, we examined the retinas of WT and Q344X rhodopsin knock-in mouse retinas for both GFAP and glutamine synthetase (GS), a marker of Müller cells (Figure 3). As predicted, Müller cells in the degenerating retinas of Q344X rhodopsin animals displayed substantially increased levels of GFAP throughout the retina. No significant measurable difference in GS labeling was observed.

### 3.5. Q344X Rhodopsin Knock-In Mouse Retinas Exhibit Apoptosis by TUNEL Labeling and Abherrant Microglial Phagocytosis

**Figure 4:**
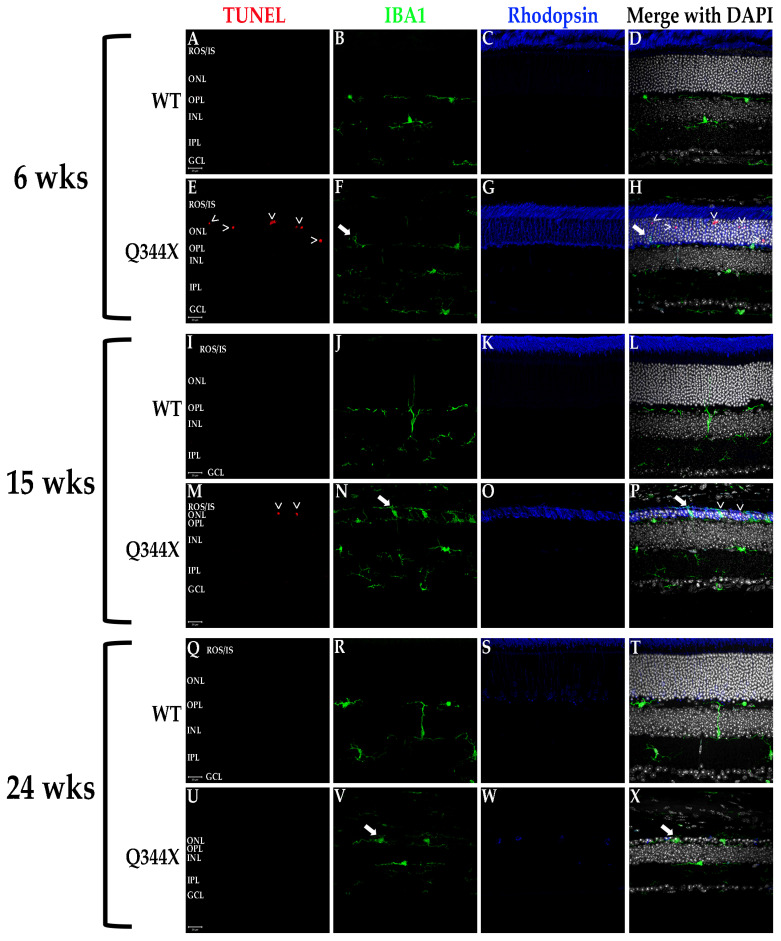
Photoreceptors undergo apoptosis while microglia proliferate and phagocytose non-apoptotic cells in the Q344X rhodopsin knock-in mouse retina. WT (**A**–**D**, **I**–**L**, **Q**–**T**) and Q344X (**E**–**H**, **M**–**P**, **U**–**X**) mouse retinal sections at 6, 15, and 24 weeks of age were TUNEL-labeled (red) and immunolabeled for IBA1 (green) and rhodopsin (blue). Nuclei were labeled with DAPI (white). ROS/IS, rod outer segments/inner segments; ONL, outer nuclear layer; OPL, outer plexiform layer; INL, inner nuclear layer; IPL, inner plexiform layer; GCL, ganglion cell layer. Arrowheads (>) indicate apoptosing cells. Arrows indicates phagoptosis (aberrant phagocytosis of living cells by microglia). Scale bars = 20 µm.

**Figure 5:**
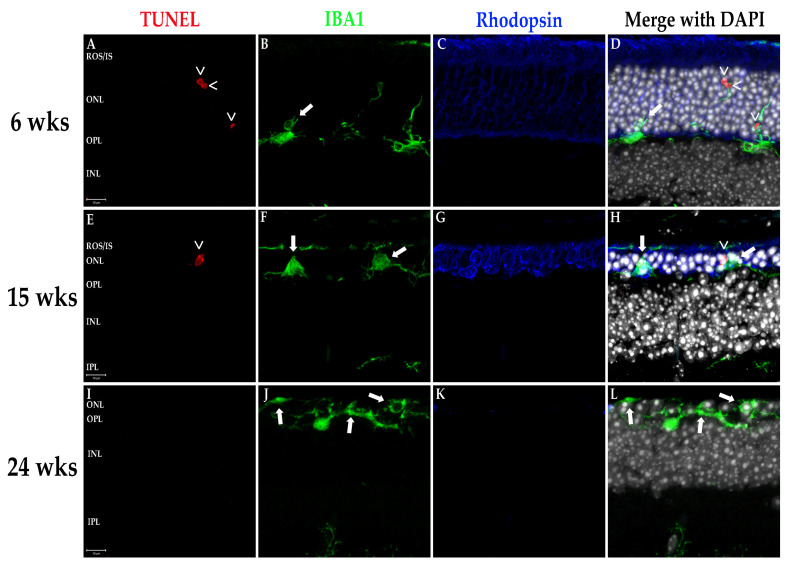
~100X magnification of TUNEL positive apoptotic cells and microglial phagoptosis of non-apoptotic cells. Q344X knock-in mouse retinal sections at 6 weeks (**A**–**D**), 15 weeks (**E**–**H**), and 24 weeks of age (**I**–**L**) were TUNEL-labeled (red) and immunolabeled for IBA1 (green) and rhodopsin (blue). Nuclei were labeled with DAPI (white). ROS/IS, rod outer segments/inner segments; ONL, outer nuclear layer; OPL, outer plexiform layer; INL, inner nuclear layer; IPL, inner plexiform layer; GCL, ganglion cell layer. Arrowheads (>) indicate apoptosing cells. Arrows indicates phagoptosis (aberrant phagocytosis of living cells by microglia). Scale bars = 10 µm.

RP progression is characterized cellularly by an escalating number of apoptotic rod photoreceptors as the diseases progresses until only cones remain, which subsequently die. The degeneration also induces activation of the resident retinal macrophages, the microglia. These cells are responsible for phagocytosing apoptotic photoreceptors and other neurons in the event of cell death as well as maintaining retinal homeostasis in conjunction with the other glial cells [17]. Under normal conditions, microglia reside in the inner retinal layers where they extend filamentous projections which sense for and detect biochemical signals originating from dying cells and/or cytokines/chemokines initiating inflammation [17,18]. Once activated, microglia migrate to the outer retina to phagocytose the apoptotic cells or foreign bodies. We evaluated retinas for photoreceptor apoptosis and microglial activity using TUNEL labeling and fIHC for IBA1, a specific marker of monocyte derived leukocytes (i.e., macrophages/microglia), respectively. As demonstrated in Figure 4, Q344X rhodopsin mice exhibit TUNEL-positive nuclei in the ONL demonstrating apoptotic death of photoreceptor cells. Also observed is a rising number of microglia migrating into the ONL, ROS/IS and RPE regions. Interestingly, microglia in these animals can be observed phagocytosing cells lacking TUNEL labeling, a phenotype observed in the RD10 mouse model of RP coined phagoptosis [19,20] (Figure 4 and Figure 5), and that this appears to occur just as often as the standard phagocytosis of apoptotic cells, if not more frequently in the fIHC performed on the retinas of these animals.

### 3.6. FIHC Labeling of the Q344X Rhodopsin Knock-In Mouse Retina Shows the Upregulation of Proinflammatory Cytokines and Pathways

**Figure 6:**
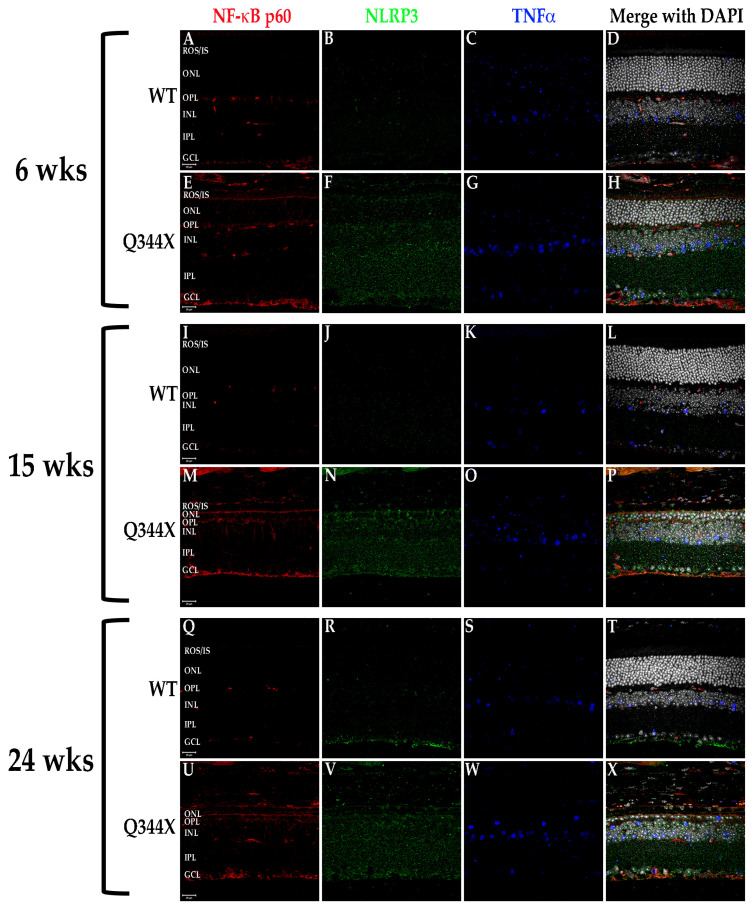
Components of the NF-kB pathway are elevated in the Q344X rhodopsin knock-in mouse retina. WT (**A**–**D**, **I**–**L**, **Q**–**T**) and Q344X (**E**–**H**, **M**–**P**, **U**–**X**) mouse retinal sections at 6, 15, and 24 weeks of age were immunolabeled for NF-kB p60 (red), NLRP3 (green) and TNFa (blue). Nuclei were labeled with DAPI (white). ROS/IS, rod outer segments/inner segments; ONL, outer nuclear layer; OPL, outer plexiform layer; INL, inner nuclear layer; IPL, inner plexiform layer; GCL, ganglion cell layer. Scale bars = 20 µm.

**Figure 7:**
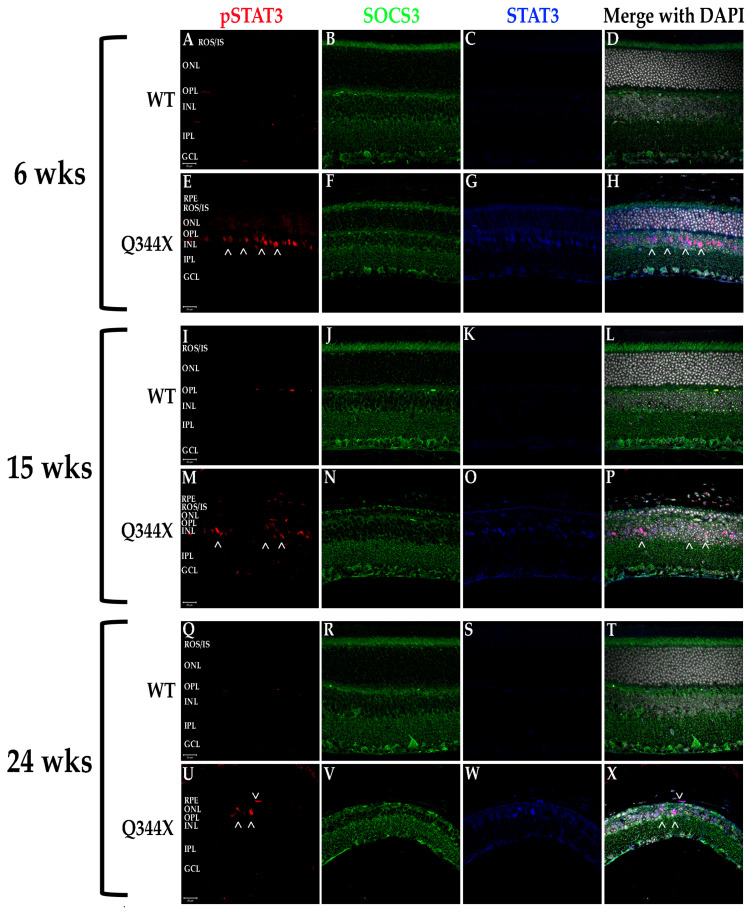
The JAK/STAT component STAT3 is upregulated and activated in the Q344X rhodopsin knock-in mouse retina. WT (**A**–**D**, **I**–**L**, **Q**–**T**) and Q344X (**E**–**H**, **M**–**P**, **U**–**X**) mouse retinal sections at 6, 15, and 24 weeks of age were immunolabeled for pSTAT3 (red), SOCS3 (green) and total STAT3 (blue). Nuclei were labeled with DAPI (white). RPE, retinal pigment epithelium; ROS/IS, rod outer segments/inner segments; ONL, outer nuclear layer; OPL, outer plexiform layer; INL, inner nuclear layer; IPL, inner plexiform layer; GCL, ganglion cell layer. Arrowheads (>) indicate regions of activated STAT3. Scale bars = 20 µm.

**Figure 8:**
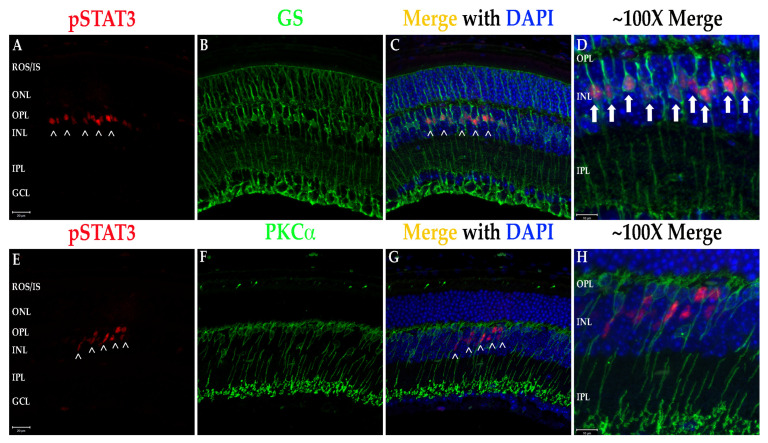
STAT3 activation in the retinal INL is confined to Müller cell nuclei. (**A**–**C**) Q344X knock-in mouse retinal sections at 6 weeks of age were immunolabeled for pSTAT3 (red) and GS (green) and imaged at 40X magnification. Nuclei labeled with DAPI (blue). Scale bar = 20 mm. Arrowheads (>) indicate regions of pSTAT3 labeling. ROS/IS, rod outer segments/inner segments; ONL, outer nuclear layer; OPL, outer plexiform layer; INL, inner nuclear layer; IPL, inner plexiform layer; GCL, ganglion cell layer. (**D**) ~100X magnification merged image. Arrows indicate complete colocalization within Müller cell nuclei. Scale bar = 10 mm. (**E**–**G**) Q344X knock-in mouse retinal sections at 6 weeks of age were immunolabeled for pSTAT3 (red) and PKCa (green). Nuclei labeled with DAPI (blue). ROS/IS, rod outer segments/inner segments; ONL, outer nuclear layer; OPL, outer plexiform layer; INL, inner nuclear layer; IPL, inner plexiform layer; GCL, ganglion cell layer. Arrowheads (>) indicate regions of pSTAT3 labeling. Scale bar = 20 mm. (**H**) ~100X magnification merged image. No colocalization with rod bipolar cell nuclei is observed. Scale bar = 10 µm.

It has been shown in recent years that retinal degenerations result in activation of multiple proinflammatory pathways including NF-κB signaling and JAK/STAT activation [3,21,22]. To test for activation of these pathways, fIHC was performed on retinal sections from WT and Q344X rhodopsin knock-in animals, targeting components of the NF-κB (TNFα, NF-κB, and NLRP3; Figure 6) and the JAK/STAT (STAT3, pSTAT3 and SOCS3; Figure 7 and Figure 8) pathways. With increasing age, retinas from Q344X rhodopsin mice have an increase in the intensity of labeling for TNFα, NF-κB, NLRP3, STAT3 and pSTAT3 compared to WT, indicating a markedly amplified proinflammatory microenvironment. It should be noted that labeling for pSTAT3 in the OPL and GCL layers is not representative of actual pSTAT3 because this labeling pattern is also observed in the no primary antibody/secondary antibody only control and is attributable to detection of endogenous mouse IgG_1_ present in the vessels of the retina (data not shown). Due to the localization of pSTAT3 in the nucleus, differentiating between the false positive labeling of blood vessels and true positive pSTAT3 labeling was performed by comparing colocalization with DAPI. In order to identify the cell type exhibiting STAT3 activation, colabeling was performed using GS or PKCα to mark Müller glia or rod bipolar cells, respectively (Figure 8). 100% INL pSTAT3 colocalizes with GS, indicating Müller glia as the pSTAT3 positive cell type.

### 3.7. Cultured Rat Müller Cells (rMC-1 Cells) Exhibit Activation of the JAK/STAT Pathway, Which Can Be Attenuated Using Pharmacological Treatment

**Figure 9:**
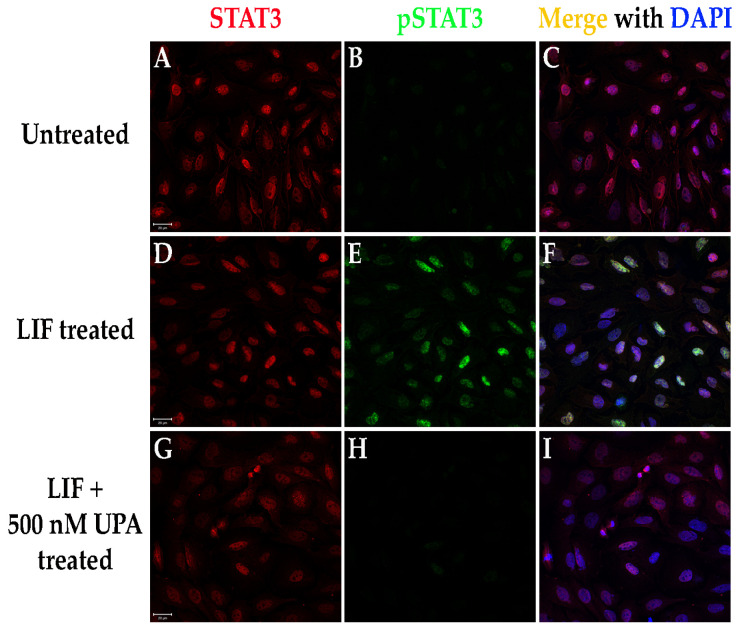
The FDA-approved pharmaceutical upadacitinib (UPA) inhibits the JAK/STAT pathway in rMC-1 cells. (**A**–**I**) rMC-1 cells were left untreated (**A**–**C**), treated using LIF (**D**–**F**), or treated using LIF with UPA (**G**–**I**) for 1 h and immunolabeled for STAT3 (red) and pSTAT3 (green). Nuclei labeled with DAPI (blue). Scale bars = 20 mm.

The ultimate goal of research into RP is a treatment or cure capable of slowing or halting the degeneration while also preserving the vision the patients still possess. Due to the strong correlations between retinal inflammation and RP progression, along with the activation of STAT3 in the Müller cells in both the Q344X and the X349E rhodopsin knock-in mouse models, we tested the hypothesis that pharmacological inhibition of the JAK/STAT pathways using an FDA-approved therapeutic can act to inhibit the JAK/STAT pathway in cultured Müller cells. To evaluate this prediction, we treated rMC-1 cells with recombinant LIF (activator of JAK/STAT signaling) either in the presence of or lacking UPA, an inhibitor of JAK activation (Figure 9). Treatment with LIF instigated activation of the JAK/STAT signaling pathway within 1 h of treatment. In the presence of UPA, pathway activation was strongly attenuated or even blocked at or slightly above relevant drug doses based on the reported IC_50_ for each JAK isoform (UPA IC_50_ = 46.8 nM JAK_1_, 120.2 nM JAK_2_ and 2.34 mM JAK_3_; DrugCentral.com).

## 4. Discussion

Many retinal degenerative diseases have a strong underlying genetic component, be it point mutations causing deleterious changes to the amino acid composition, structure and function of the protein product coded for by the gene or single-nucleotide polymorphisms (SNPs) which can also affect the regulatory mechanisms governing gene expression. While the exerted effects of these genetic alterations may initiate retinal degeneration, the progression of the degeneration has another factor that heavily influences it—the immune system. In the last 10 to 15 years, multiple labs have explored the previously underscored role of innate and autoimmunity in degenerative progression. All retinal degenerative diseases have been found to be linked to dysregulation of the ocular immune environment in one facet or another. AMD risk is heavily correlated with SNPs in most the genes associated with the complement system [23,24] while also having accumulation of immune components in the drusen deposits associated with AMD [25,26]. Glaucoma has been associated with autoantibody production to heat shock proteins [27,28,29] and increased presence of microglia [17,30]. Even diabetic retinopathy, a retinal degenerative disease which can have no genetic component if associated with type 2 diabetes, has been shown in multiple studies to result in proinflammatory cytokines and chemokines such as TNFα, monocyte chemoattractant protein-1 and transforming growth factor-β to be secreted and microglial activation/infiltration of bloodborne macrophages into the retina from vascular leakage [31,32,33,34].

In RP, we and other labs have shown in multiple models the upregulation of proinflammatory cytokines, increased microglia in the subretinal space and activation of proinflammatory pathways [3,8,10]. Due in part to the complex nature of the disease pathogenesis of not only RP, but also AMD and glaucoma, treatments for these diseases are lacking. Leber’s congenital amaurosis (LCA), a much earlier onset and faster progressing form of RP, has only recently received effective treatments in the form of gene therapy to replace the defective gene associated with the disease; however, these therapies do not work for all the genes associated with the diseases (LCA has an FDA-approved gene therapy for supplementing the gene for retinal pigment epithelium 65 kD protein, or RPE65) [35,36]. In fact, treating the autosomal dominant form of RP has proven to be challenging as simply supplementing back the defective gene will not salvage the degenerative phenotype due to the deleterious effect the mutant protein elicits on cell survival [37]. As an example, mutated rhodopsin ultimately causes defective functioning of normal rhodopsin by either mislocalizing it to the inner segments and synapses of the rod cell, loss or gain-of-function, or causing its retention in the endoplasmic reticulum [1,2,38].

Due to the heterogenous nature of retinal degenerative diseases, having treatments that modulate phenotypes observed in all forms would have the most impact on vision preservation in patients. Thus, it is only logical that immunomodulating treatments would be highly likely to slow degeneration in these diseases. In this work, we uncovered a similar immunological phenotype as we observed in the X349E human rhodopsin knock-in mouse model; however, we expanded on those phenotypes by further examining other proinflammatory pathways and the components associated with them within the Q344X rhodopsin mouse retina. TNFα is a well-known cytokine capable of initiating the NF-κB signaling pathway and ultimate upregulation of the NLRP3 inflammasome [10,31]. According to our data, not only do we see an increase in the amount of TNFα present, but also a strong upregulation of NF-κB expression and shift in localization and a similar increase in the NLRP3 present throughout the retina. Similar to the X349E rhodopsin knock-in retina, we see a dramatic increase in STAT3 phosphorylation and most of this increase is localized to the INL. The X349E knock-in retina exhibited localization to the INL and that localization was traced to Müller cells and, indeed, the Q344X retina exhibits similar Müller cell localization of pSTAT3 [3]. In contrast, however, no real SOCS3 changes occurred in the Q344X knock-in retina based on mean intensity analysis of fIHC images.

With all retinal degenerations, slowing the degeneration not only maintains a better quality of life for the patients by prolonging their sight, but also allows for more time for researchers to develop more effective treatments allowing for permanent vision restoration and maintenance, such as gene therapy. As such, our work presented here opens the door for testing immunomodulating small molecule delivery to the retinas of these mice through topical application utilizing our group’s expertise in small molecule therapeutics, microemulsion formulations and drug delivery systems [39,40,41,42]. We began such testing of possible pharmaceuticals in this study using UPA, a high-affinity potent JAK inhibitor, to try and inhibit the JAK/STAT signaling pathway in rMC-1 rat Müller cells as this cell type displayed the most labeling for pSTAT3 in both the Q344X and X349E mouse retinas. We observed that UPA is capable of strongly inhibiting the JAK/STAT pathway in LIF induced rMC-1 cells, an indication to us that this drug and inhibition of the JAK/STAT pathway could be beneficial in the treatment of RP and other retinal degenerations. Our future work will include testing formulations of this and other drugs in our own microemulsion technology for better and longer-lasting delivery of immunomodulatory compounds to the retina, bypassing systemic administration and, hopefully, provide a treatment to the vision world for slowing the progression of these devastating blinding diseases.

## Figures and Tables

**Table 1:** Antibodies used for IHC labeling of retinal sections.

Antibody Target	Host Species and IgG Isoform	Dilution Factor	Antibody Source
GFAP	Rabbit IgG	1:200	ProteinTech
GS	Mouse IgG_2a_	1:500	EMD Millipore
IBA1	Rabbit IgG	1:250	Cell Signaling Tech.
NF-κB	Rabbit IgG	1:400	Cell Signaling Tech.
NLRP3	Rat IgG_2a_	1:250	ThermoFisher
pSTAT3	Mouse IgG_1_	1:100	Cell Signaling Tech.
PKCα	Mouse IgG_2a_	1:250	ThermoFisher
RHO (B6-30N)	Mouse IgG_1_	1:500	C/O: W. Clay Smith
SOCS3	Rabbit IgG	1:100	abcam
STAT3	Mouse IgG_2a_	1:100	Cell Signaling Tech.
TNFα	Mouse IgG_2b_	1:250	ProteinTech

## Supplementary Materials

The following are available online at https://www.mdpi.com/article/10.3390/biom11081163/s1, Supplementary Figure S1: Q344X rhodopsin knock-in mouse retinas degenerate rapidly by OCT; Supplementary Figure S2: Western blot analyses of quantitated fIHC probed proteins.; Supplementary Figure S3: TUNEL positive apoptotic cells and microglial phagoptosis of non-apoptotic cells.; Supplementary Figure S4: Quantification of fIHC for inflammatory pathway components.
