# Supplementary material for: Proinflammatory Pathways Are Activated in the Human Q344X Rhodopsin Knock-In Mouse Model of Retinitis Pigmentosa
Source: Biomolecules. 2021 Aug 6;11(8):1163. doi: 10.3390/biom11081163 (PMC8393353; doi:10.3390/biom11081163)
Supplement: Supplementary file 1 [file biomolecules-11-01163-s001.zip › biomolecules-1236213-supplementary.pdf]

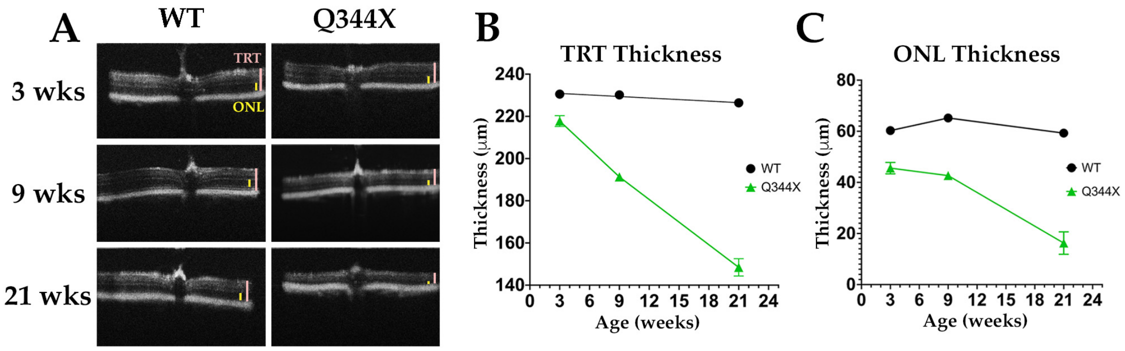

Supplementary Figure 1. Q344X rhodopsin knock-in mouse retinas degenerate rapidly by OCT. A) OCT images of WT and Q344X rhodopsin knock-in mouse retinas through the optic nerve head at 3, 9, and 21 weeks of age. ONL thickness shown by yellow caliper. Total retinal thickness (TRT) shown by pink caliper. **B)** Graphical representation of TRT (WT, black circles; Q344X, green triangles). **C)** Graphical representation of ONL thickness (WT, black circles; Q344X, green triangles). Error bars = ±SD.

Figure S1. Q344X rhodopsin knock-in mouse retinas degenerate rapidly by OCT.

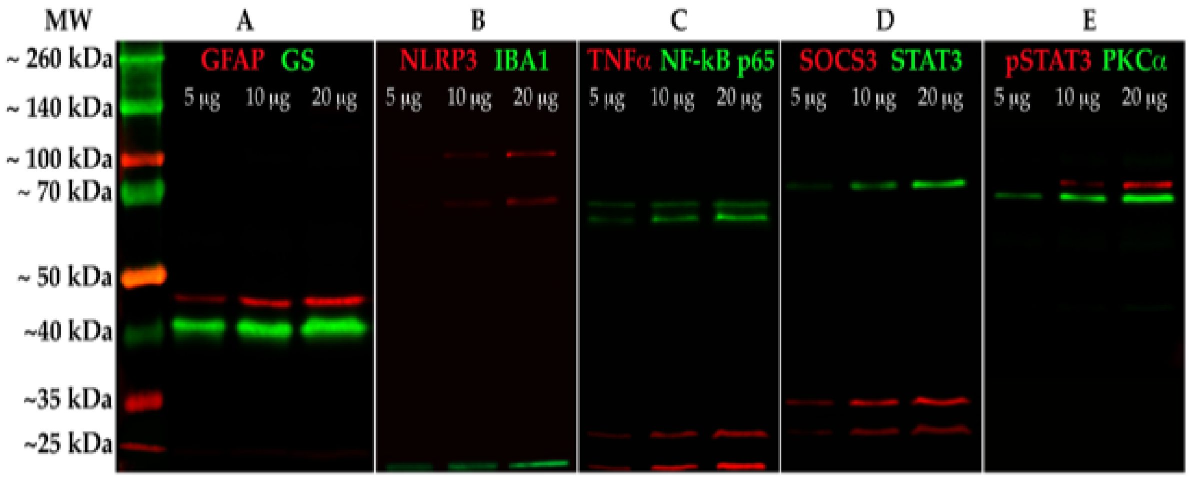

Supplementary Figure 2. Western blot analyses of quantitated fIHC probed proteins. Q344X mouse retinas at 6 weeks of age were extracted, sonicated in RIPA buffer, and 5, 10, and 20 µg of protein electrophoresed in sequential wells. Blots were probed with the same antibodies used for fIHC to confirm specificity. **A)** GFAP (red), GS (green); **B)** NLRP3 (red), IBA1 (green); **C)** TNFα (red), NF-κB p65 (green); **D)** SOCS3 (red), STAT3 (green); **E)** pSTAT3(red), PKCα (green). MW = molecular weight in kiloDaltons.

Figure S2. Western blot analyses of quantitated fIHC probed proteins.

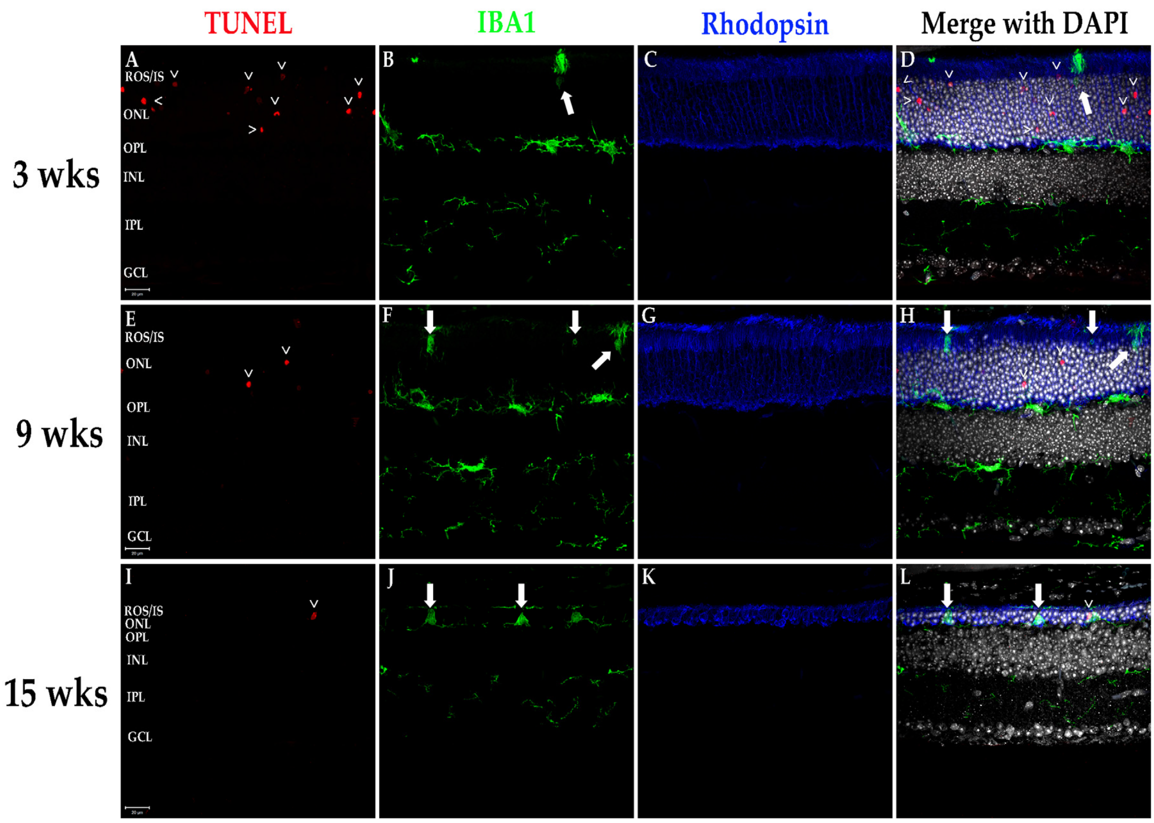

Supplementary Figure 3. TUNEL positive apoptotic cells and microglial phagoptosis of non-apoptotic cells. Q344X knock-in mouse retinal sections at 3 weeks (A-D), 9 weeks (E-H), and 15 weeks of age (I-L) were TUNEL-labeled (red) and immunolabeled for IBA1 (green) and rhodopsin (blue). Nuclei were labeled with DAPI (white). ROS/IS, rod outer segments/inner segments; ONL, outer nuclear layer; OPL, outer plexiform layer; INL, inner nuclear layer; IPL, inner plexiform layer; GCL, ganglion cell layer. Arrowheads (>) indicate apoptosing cells. Arrows indicates phagoptosis (aberrant phagocytosis of living cells by microglia). Scale bars = 20 µm.

Figure S3. TUNEL positive apoptotic cells and microglial phagoptosis of non-apoptotic cells.

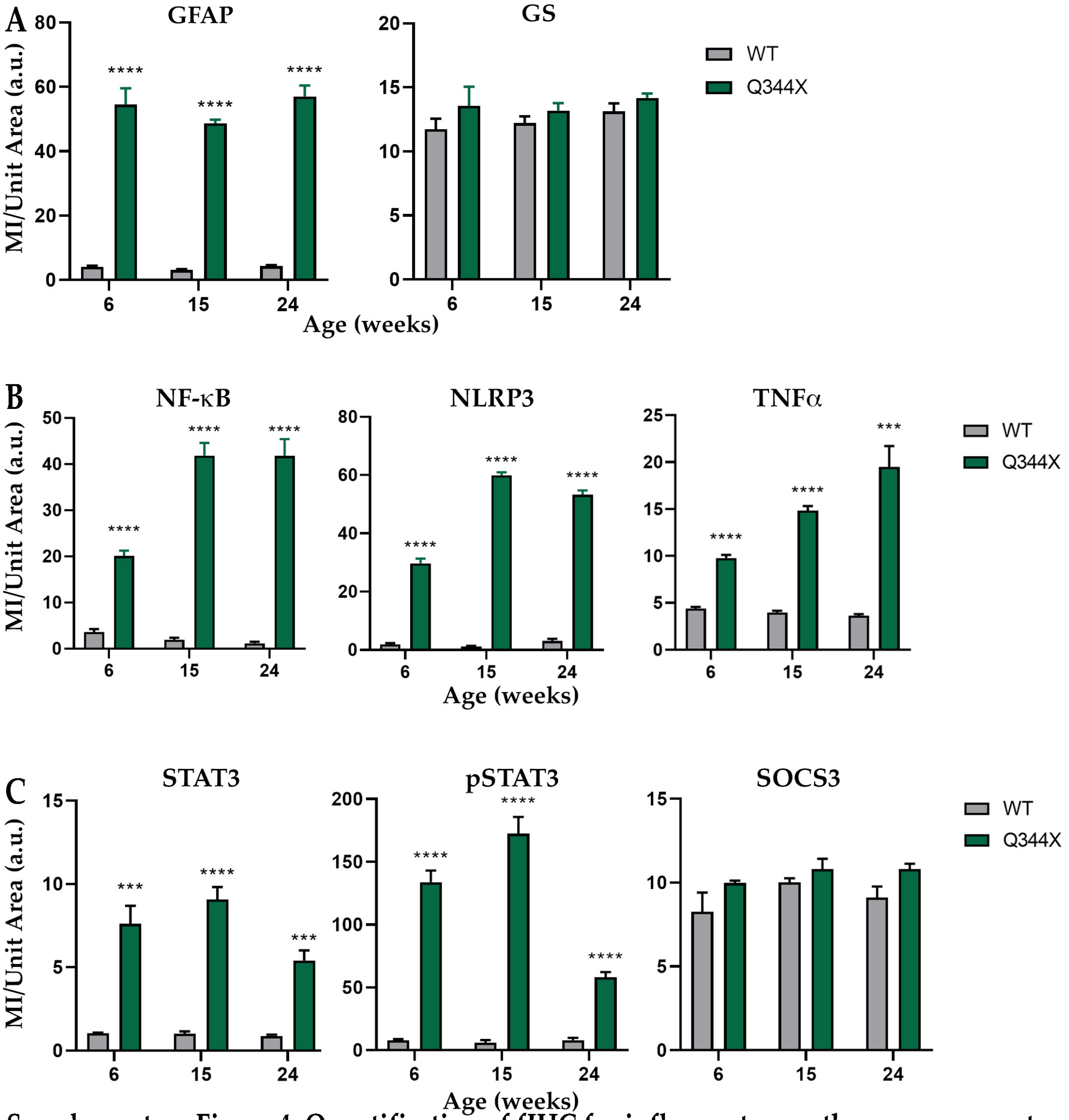

Supplementary Figure 4. Quantification of fIHC for inflammatory pathway components. Using ImageJ, mean intensity (MI) per unit area in arbitrary units (a.u.) was assessed for fIHC labeling of GFAP and GS (A); NF-κB, NLRP3, and TNFα (B); and STAT3, pSTAT3, and SOCS3 (C) in WT (grey) and Q344X (green) mouse retinas. Error bars = ±SEM. ***, p<0.0001; ****, p<0.00001.

Figure S4. Quantification of fIHC for inflammatory pathway components.
